# The Association of Serum Leptin with Mortality in Older Adults

**DOI:** 10.1371/journal.pone.0140763

**Published:** 2015-10-16

**Authors:** Suruchi Mishra, Tamara B. Harris, Wen-Chi Hsueh, Trisha Hue, Tennille S. Leak, Rongling Li, Mira Mehta, Christian Vaisse, Nadine R. Sahyoun

**Affiliations:** 1 Department of Nutrition and Food Science, University of Maryland, College Park, Maryland, United States of America; 2 National Institute on Aging, National Institute of Health, Bethesda, Maryland, United States of America; 3 Phoenix Epidemiology and Clinical Research Branch, National Institute of Diabetes and Digestive and Kidney Diseases, National Institute of Health, Phoenix, Arizona, United States of America; 4 University of California San Francisco, San Francisco, California, United States of America; 5 Michigan Public Health Institute, Okemos, Michigan, United States of America; 6 National Human Genome Research Institute, National Institute of Health, Rockville, Maryland, United States of America; 7 Diabetes Center and Department of Medicine, University of California San Francisco, San Francisco, California, United States of America; University of Catanzaro Magna Graecia, ITALY

## Abstract

**Objective:**

Elevated levels of serum leptin are associated with increased adiposity and production of pro-inflammatory cytokines. Both cytokines and body adiposity have been shown to predict cardiovascular events and mortality. The primary objective of the present study is to explore the associations between serum leptin and all-cause mortality and mortality from cardiovascular disease (CVD) over a span of 10 years, controlling for body adiposity and proinflammatory cytokines.

**Methods:**

The Health, Aging and Body Composition (Health ABC) study is a prospective cohort of 3,075 older adults aged 70 to 79 years. This analysis includes 2,919 men and women with complete serum leptin and vital status data. Data on all-cause mortality and incident cardiovascular events (including Coronary Heart Disease and Congestive Heart Failure) were collected over 10 years of follow-up (mean 8.4 years).

**Results:**

Women with leptin in quartile 2 and 3 were at lower risk of all-cause mortality, and those with leptin in quartile 2 were at lower risk of mortality from CVD as compared to women with lowest leptin values when adjusted for age, race, site, years of education, alcohol use, smoking, and physical activity. When these associations were additionally adjusted for body fat, C-reactive protein and pro-inflammatory cytokines, women with leptin values in quartile 3 were at lower risk of all-cause mortality and women with leptin in quartile 2 and 3 were at lower risk of mortality from CVD than women with lowest leptin values. These associations were not significant among men after adjusting for body fat and cytokines.

**Conclusions:**

The present study suggests that moderately elevated concentrations of serum leptin are independently associated with lower risk of all-cause mortality and CVD-related mortality among older women. Among men, serum leptin is not associated with reduced risk of all-cause and CVD mortality after controlling for body fat and cytokines.

## Introduction

Leptin plays a critical role in energy intake [1, 2], inflammatory response [3], and is strongly correlated with body fat [3, 4, 5, 6], which is linked to mortality [7, 8, 9]. Serum leptin and proinflammatory cytokines have been associated in the development of several age-associated conditions such as metabolic syndrome and atherosclerosis [7, 10, 11, 12, 13, 14, 15]. With aging, there is a concomitant increase in the levels of serum leptin, proinflammatory cytokines [3, 7, 16, 17, 18] that are linked to obesity [1, 19], and the prevalence of metabolic diseases [1, 14, 20, 21, 22, 23, 24]. Leptin and its receptor have structural and functional similarities with the interleukin-6 (IL-6) family of cytokines [13] and high levels of IL-6 were associated with cardiovascular and other causes of mortality among healthy older adults [7]. Increases in tumor necrosis factor- α (TNF- α) and serum leptin levels with aging have been associated with obesity and atherosclerosis [25, 26, 27, 28]. Previous studies on the role of leptin on cardiovascular function have shown conflicting results. Leptin exerts protective effect on cardiovascular function by modulating nitric oxide synthesis in human endothelial cell culture study [29]. However, hyperleptinemia induces chronic oxidative stress in endothelial cells and activates atherogenic process *in vitro* models [17]. In some population based studies, elevated levels of leptin have been shown to be associated with increased risk of metabolic syndrome [24, 15] and incident coronary artery disease [23], while others found no association [30, 31].

Although serum levels of leptin have been consistently shown to be associated with increased inflammation and morbidity, the association of leptin with survival has not been well-studied independently of inflammatory factors and body adiposity among functionally well older adults.

The primary objective of this analysis was to examine the association between serum leptin and the risk of all-cause mortality and CVD-related mortality over a 10-year follow-up and to investigate whether these associations are independent of markers of inflammation and body fat.

## Methods

The study is secondary data analysis and was approved by IRB Office, University of Maryland, College Park, Lee Building, Room 2100, Zip 5125, College Park, Maryland 20742. IRB APPLICATION # 09–0527. Written informed consent was given by participants in the Health ABC study for their clinical records to be used.

### Study Design

The Health ABC study is a longitudinal cohort study of 3,075 community-dwelling, well-functioning white and black men and women aged 70 to 79 years at the commencement of the study. This study was described in details elsewhere [32]. Briefly, participants were recruited for the study from a random sample of white residents receiving Medicare benefits and all age-eligible black residents of Pittsburgh, Pennsylvania, and Memphis, Tennessee. The subjects were considered eligible to participate in the study if they reported no difficulty walking a quarter of a mile, climbing 10 steps without resting, or performing basic activities of daily living, were free of life-threatening illness, and planned to remain in the geographic area for at least 3 years. Those who reported active treatment for cancer or participation in diet or exercise intervention were excluded from the study. Participants gave written informed consent, and protocols were approved by the Institutional Review Boards at the two study sites. The participants’ information was anonymized and de-identified prior to analysis. Enrollment in the cohort and baseline clinic visits, which included collection of serum and measures of body composition, weight-related health conditions, and physical function, were conducted between April 1997 and June 1998. Thereafter, clinical examinations to obtain follow-up body composition measures and clinical outcome data were conducted annually for six years, and then every other year through year 10. Semi-annual phone interviews were also conducted between clinic visits to ascertain outcome data,

### Subjects

In the present analysis, data from baseline through year 10 of the Health ABC study were used. The sample size for this analysis was 2,919 (men = 1,419; women = 1,500) after excluding those with incomplete information for vital status (n = 101) and missing serum leptin (n = 55). One hundred thirteen (n = 113) participants were missing data for total percent body fat, 119 for abdominal visceral fat, 209 for abdominal subcutaneous fat, 203 for TNF-α, 38 for C-reactive protein (CRP), 65 for plasminogen activator inhibitor-1(PAI-1) and 162 for IL-6. The number of participants missing information for these variables did not differ by the outcome variable.

### Baseline Biochemical Markers

Blood samples were collected in the morning when participants underwent venipuncture at the baseline visit after an overnight fast, and serum samples were frozen at -70°C. Both IL-6 and TNF- α were measured in duplicate using an ultrasensitive enzyme-linked immunosorbent assay (R&D Systems, Minneapolis, MN, USA). The limit of detection was 0.10 pg/ml for IL-6 and 0.18 pg/ml for TNF- α. The level of PAI-1 was measured in citrated plasma samples using a 2-site enzyme-linked immunosorbent assay. Serum levels of CRP were also measured in duplicate by enzyme-linked immunosorbent assay based on purified protein and polyclonal anti-CRP antibodies (Calbiochem, San Diego, CA, USA) with a coefficient of variation of 8.0%. Serum concentrations of leptin were measured in duplicate by means of radioimmunoassay (Linco Research Inc, St Charles, MO, USA). The assay range is 0.05 to 100 ng/ml leptin in serum. Intra-assay CVs ranged from 3.7% to 7.5%, and inter-assay CVs ranged from 3.2% to 8.9%.

### Baseline Anthropometric Variables

Weight was measured in kilograms using a standard balance beam scale. Height was measured twice in centimeters using a Harpenden stadiometer (Holtain Ltd., Crosswell, U.K.) and the average of the two measurements was used. BMI [weight (kg)/height (m^2^)] was calculated. Dual energy x-ray absorptiometry (DXA) (Hologic QDR 4500A, software version 8.21, Hologic, Waltham, MA, USA) was used to assess total fat mass and total percentage of body fat was calculated.

### Demographic and Lifestyle Variables

A standardized questionnaire was administered at baseline to collect information on socio-demographic variables including age, gender, self-identified race, years of education, and lifestyle variables including smoking status and average alcohol consumption during the past year. Information on use of estrogen or female hormone pills (Hormonal Replacement Therapy, HRT) since menopause was also collected from women. Cigarette packs smoked per day were multiplied by the number of years of smoking to calculate the pack-years over a lifetime of cigarette smoking. Level of physical activity was ascertained by a standardized questionnaire specifically designed for the Health ABC study [33]. The frequency, duration, and intensity level of activities such as self-report of walking and exercise were recorded, and approximate values of metabolic equivalent unit (MET) were assigned to each activity category to estimate weekly energy expenditure in kcal/kg/week. Dietary calorie intake was measured during Year 2 of the Health ABC Study, at the first annual follow-up visit using a 108-item food frequency questionnaire (FFQ) that was designed to assess intake in the year prior. Nutrient and food group intakes were determined by Block Dietary Data Systems [34].

### Outcome Variables

All deaths were adjudicated by a central committee. The outcomes included in this analysis were all-cause mortality, and CVD-related mortality events occurring between baseline through November 26, 2007. Every 6 months, participants either received an in-person examination or were contacted by telephone. Deaths were also ascertained through review of death certificates, hospital records, proxy interviews, and Social Security Death Index data. All deaths in Health ABC were adjudicated by a central committee. CVD-related death was defined as any death where the underlying cause was confirmed as atherosclerotic cardiovascular disease (definite fatal myocardial infarction, or definite or possible fatal coronary artery disease), cerebrovascular disease, atherosclerotic disease other than cardiovascular, and other cardiovascular disease.

### Statistical Analysis

Serum leptin values and body fat vary by gender; therefore, the analysis was performed separately by gender [15, 35]. Baseline characteristics of men and women were examined by quartiles of serum leptin. For continuous variables, least square means were computed with Dunnett’s test option, and for categorical variables, the chi-square test was used to compare the means of quartile 2 through 4 to those of quartile 1. The inflammatory markers and leptin was log transformed because they were not normally distributed. The correlations between explanatory variables (age, smoking status, exercise, total percent fat, leptin, CRP, IL-6, TNF- α, and PAI-1) were examined and multicollinearity was assessed by using variance inflation factors (VIF). The VIF remained below the suggested cut off value of four for all explanatory variables. Interactions between serum leptin with race, inflammatory markers (CRP, IL-6, TNF- α, and PAI-1), adiposity measures (total percent fat) and CVD diagnosis were tested; none were statistically significant based on alpha level of 0.05.

Survival time was defined as the time from the date of the baseline visit until the date of death and/or date of last contact. Cox proportional hazard regression analysis was used to assess the risk of all-cause mortality and mortality from CVD for persons in quartiles 2 through 4 of serum leptin with those in quartile 1. The regression models were sequentially adjusted for potential confounders (age, gender, race, study site, education, smoking status, alcohol use, physical activity, HRT use, and number of hours fasted), total percent fat and markers of systemic inflammation (CRP, TNF- α, IL-6 and PAI-1). Final model included the main predictor variable (leptin) and covariates that were significantly associated with the outcome. Log transformed leptin was used in linear regression model for trend analysis. All the covariates met the assumptions for proportional hazard regression.

Statistical significance was set at p ≤ 0.05, and analysis was performed using SAS software program (version 9.1; SAS Institute Inc., Cary, NC).

## Results

There was a significant difference (p<0.0001) between the mean serum leptin concentrations of men 7.9 (±6.9) ng/ml and women 21.3 (±14.6) ng/ml. Baseline characteristics indicate that a lower percentage of men and women in quartile 4 of serum leptin consumed alcohol than those in quartile 1. Men in quartile 3 were more likely to be white and have completed high school, as compared to those in quartile 1. Women in quartile 2, 3, and 4 were less likely to be white and those in quartile 3 and 4 were less likely to complete high school as compared to those in quartile 1. As expected, men and women with leptin in quartile 1 had significantly lower BMI and total percent body fat as compared to those with higher serum leptin,Tables 1 and 2. Women in quartile 1 had significantly lower CRP and IL-6 levels than those in quartile 3 and 4. Individuals (men and women) with leptin in quartile 1 had significantly lower TNF-α than those in quartile 4 and significantly lower PAI-1 than those in quartiles 2, 3, and 4.

**Table 1:** Baseline characteristics of men by serum leptin quartile^a^.

	Serum Leptin Quartile			
	1	2	3	4
**Men (n)**	354	355	355	355
Mean Serum Leptin (ng/ml)^c^	2.11 (±0.93)	4.77 (±0.76)	7.92 (±1.13)	16.97 (±8.13)
**Demographic and behavioral variables**				
Age (years)^c^	75.4 (±2.9)	75.1 (±2.5)	75.4 (±3.0)	75.2 (±2.8)
Race (% white) ^c^	62	67	69^b^	56
Alcohol use (% any consumption) ^c^	64	60	55	53^b^
Education (% completed high school) ^c^	69	73	76^b^	74
Smoking (lifetime pack-years)^c^	25.2 (±29.6)	25.2 (±29.5)	25.4 (±31.9)	27.1 (±31.1)
Physical activity (kcal/kg/week) ^c^	9.35 (±20.6)	8.8 (±20.9)	7.7 (±17.1)	7.4 (±15.5)
**Dietary and anthropometric variables**				
Total calorie intake (kcal)^d^	2128 (±867)	2084 (±849)	1991 (±798)	1875 (±775)
BMI (kg/m^2^)^c^	23.75 (±2.7)	26.1 (±2.8)^b^	28.1 (±2.9)^b^	30.2 (±3.9)^b^
Total body fat (%)^c^	24.1 (±3.8)	28.2 (±3.0)^b^	31.2 (±3.4)^b^	33.3 (±4.2)^b^
**Biochemical markers**				
C-reactive protein (μg/ml) ^c^	2.44 (±4.3)	2.7 (±5.0)	2.7(±5.4)	2.8 (±3.1)
IL-6 (pg/ml)^c^	2.46 (±2.1)	2.27 (±1.62)	2.55 (±2.1)	2.6 (±1.8)
TNF-alpha (pg/ml)^c^	3.5 (±1.8)	3.5 (±2.1)	3.6 (±1.5)	3.9 (±1.9)^b^
PAI-I ^c^	22.6 (±1.0)	28.1 (±1.1)^b^	28.1 (±1.1)^b^	27.5 (±1.3)^b^

^a^ Means (±SEM), unless otherwise specified.

^b^ Significantly different from leptin quartile 1, P≤0.05 (Dunnett’s test for continuous variables and chi-square test for categorical variables).

^c^ Values from baseline of the Health ABC study.

^d^ Values from year 2 of the Health ABC study.

**Table 2:** Baseline characteristics of women by serum leptin quartile^a^.

	Serum Leptin Quartile			
	1	2	3	4
**Women (n)**	375	375	375	375
Mean Serum Leptin (ng/ml)^c^	6.4 (±2.6)	14.0 (±2.2)	22.7 (±2.9)	42.1 (±11.1)
**Demographic and behavioral variables**				
Age (years)^c^	75.3 (±2.9)	75.3 (±2.9)	75.0 (±2.8)	75.0 (±2.8)
Race (% white) ^c^	71	59^b^	51^b^	61^b^
Alcohol use (% any consumption) ^c^	45	47	44	35^b^
Education (% completed high school) ^c^	84	78	77^b^	70^b^
Smoking (lifetime pack-years)^c^	14.2 (±25.2)	10.6 (±18.7)	13.4 (±25.6)	11.2 (±21.8)
Physical activity (kcal/kg/week)^c^	5.1 (±11.8)	4.6 (±10.3)	2.9 (±7.5)^b^	3.9 (±15.6)
HRT use (% yes) ^c^	41	44	42	38
**Dietary and anthropometric variables**				
Total calorie intake (kcal)^d^	1731 (±627)	1717 (±684)	1701 (±657)	1677 (±629)
BMI (kg/m2)^c^	23.1 (±3.7)	26.4 (±3.9)^b^	29.0 (±4.3)^b^	32.3 (±4.9)^b^
Total body fat (%)^c^	35.0 (±5.4)	39.5 (±3.9)^b^	42.2 (±3.7)^b^	45.0 (±4.1)^b^
**Biochemical markers**				
C-reactive protein (μg/ml)^c^	2.6 (±5.6)	2.6 (±2.4)	3.7 (±4.6)^b^	4.3 (±5.2)^b^
IL-6 (pg/ml)^c^	2.0 (±1.8)	1.9 (±1.5)	2.5 (±2.1)^b^	2.8 (±2.0)^b^
TNF-alpha (pg/ml)^c^	3.3 (±1.6)	3.3 (±1.5)	3.3 (±1.3)	3.7 (±1.9)^b^
PAI-I ^c^	21.9 (±1.1)	27.6 (±1.2)^b^	33 (±1.2)^b^	36.5 (±1.5)^b^

^a^ Means (±SEM), unless otherwise specified.

^b^ Significantly different from leptin quartile 1, P≤0.05 (Dunnett’s test for continuous variables and chi-square test for categorical variables).

^c^ Values from baseline of the Health ABC study

^d^ Values from year 2 of the Health ABC study

Serum leptin was significantly correlated with age, race, site, smoking, alcohol use (p<0.001, r ranged from -0.14 to 0.12), total percent body fat (p<0.0001, r = 0.79), cytokines (TNF- α, IL-6, and PAI-1) (p<0.001, r ranged from 0.04 to 0.27) and CRP (p<0.0001, r = 0.24). These variables were included in the multivariable models.

The average follow-up time from baseline was 8.4 years, with a range of 1.1 to 10.4 years. During the follow-up time, 556 (39.2%) men and 401 (26.7%) women were deceased. Among men, there was no significant association between serum leptin and all-cause mortality. Among women, those with serum leptin values in quartile 2 (HR = 0.71; 95% CI = 0.53, 0.95) and 3 (HR = 0.73; 95% CI = 0.55, 0.98) had significantly lower risk of mortality as compared to those in quartile 1 after adjusting for age, race, site, years of education, alcohol use, smoking, and physical activity in model 1. With further adjustment for total percent fat and proinflammatory cytokines, the association between quartile 3 of serum leptin and all-cause mortality remained marginally significant (HR = 0.68; 95% CI = 0.47, 0.99). As indicated by the trend analysis, the association between leptin and mortality was not linear. No associations were found among men, Table 3. During follow-up, 197 (13.9%) men and 154 (10.3%) women died due to mortality from CVD. There were no significant associations between risk for CVD- related mortality and serum leptin levels among men. Women with leptin levels in quartile 2 (HR = 0.60; 95% CI = 0. 37, 0.97) were at lower risk for CVD- related mortality, as compared to women with leptin levels in quartile 1 after adjustment for potential confounders. When percent body fat and inflammatory markers were added to the model, women with leptin values in quartile 2 and 3 were at lower risk for mortality from CVD (HR = 0.52; 95% CI = 0. 28, 0.97 and HR = 0.44; 95% CI = 0. 22, 0.89, respectively) as compared to those with leptin values in lowest quartile. No linear associations were found between leptin and the risk of mortality from CVD among men and women, Table 3.

**Table 3:** Hazard ratios (HR) for all-cause mortality and CVD-related mortality by serum leptin quartile.

Serum leptin quartiles	1	2	3	4	P for trend^a^
**All-cause mortality**					
**Men (n)**	354	355	355	355	
All-Cause mortality cases (n)	151	139	125	141	0.241
All-Cause mortality (%)	43	39	35	40	
Model 1^b^					
HR (95% CI)	1	0.97 (0.76–1.23)	0.79 (0.61–1.01)	0.88 (0.69–1.12)	0.44
Model 2^c^					
HR (95% CI)	1	0.93 (0.70–1.23)	0.74 (0.53–1.03)	0.82 (0.56–1.94)	0.06
**Women (n)**	375	375	375	375	
All-Cause mortality cases (n)	111	92	95	103	0.0003
All-Cause mortality (%)	30	25	25	27	
Model 1^b^					
HR (95% CI)	1	0.71 (0.53–0.95)	0.73 (0.55–0.98)	0.84 (0.63–1.13)	0.50
Model 2^c^					
HR (95% CI)	1	0.72 (0.51–1.02)	0.68 (0.47–0.99)	0.76 (0.50–1.67)	0.89
**CVD-related mortality**					
**Men (n)**	354	355	355	355	
CVD-related mortality cases (n)	45	53	44	55	0.48
CVD-related mortality %	12.7	14.9	12.4	15.5	
Model 1^b^					
HR (95% CI)	1	1.96 (0.79–1.83)	0.99 (0.64–1.53)	1.09 (0.71–1.67)	0.21
Model 2^c^					
HR (95% CI)	1	0.99 (0.61–1.60)	0.71 (0.40–1.23)	0.67 (0.35–1.27)	0.14
**Women (n)**	375	375	375	375	
CVD mortality cases (n)	44	36	35	39	0.543
CVD-related mortality %	11.7	9.6	9.3	10.4	
Model 1^b^					
HR (95% CI)	1	0.60 (0.37–0.97)	0.71 (0.45–1.25)	0.76 (0.48–1.21)	0.71
Model 2^c^					
HR (95% CI)	1	0.48 (0.27–0.86)	0.50 (0.27–0.92)	0.59 (0.29–1.19)	0.85

^a^ Tests for linear trend used leptin as a continuous variable in logistic regression.

^b^ Model 1: Adjusted for age, race, site, years of education, alcohol use, smoking, and physical activity (total calorie intake, HRT use among women and numbers of hours fasted were not significant and did not affect the association between predictor and outcome, so they were not included in the model)

^c^ Model 2: Adjusted for variables in model 1 plus TOTPF, CRP, PAI-1, TNF, and IL6

## Discussion

In the present study intermediate levels of serum leptin were associated with lower risk of all-cause mortality and CVD- related mortality. These associations remained significant after controlling for measures of body adiposity and the pro-inflammatory cytokines.

Results of this study are supported by previous studies that have shown an association between serum leptin and all-cause and CVD mortality [8, 17, 36]. Lieb et al., in Framingham Heart Study found a U-shaped association between serum leptin and all-cause mortality with elevated risk of death at both low and high levels of leptin among older men and women [36]. Furthermore, they showed that the U-shape association was mainly contributed by increased risk of death due to non-cardiovascular diseases [36]. Hypoleptinemia has been shown to elevate the risk of cancer mortality [37], while hyperleptinemia increased the risk of mortality from type 2 diabetes [38]. In a longitudinal study of impaired glucose-tolerant and diabetic women aged 49 to 73 years, CVD mortality significantly decreased in intermediate and higher tertiles as compared to lowest tertile of serum leptin concentrations [27]. In the Heart and Soul Study, low leptin was associated with 37% increased risk of cardiovascular events in patients with mean age of 67 years and stable coronary artery disease after adjusting for obesity and traditional cardiovascular risk factors [8]. In our study, we investigated gender specific associations of leptin with risk of all-cause and CVD-related mortality among functionally well older adults. Our results of an inverse association between leptin levels and all-cause and CVD mortality at moderately elevated, but not at high, levels of leptin among older women are consistent with previous reports.

Additionally, we showed that this association remained significant after controlling for body fat and cytokines among older women but not among older men. Studies have consistently shown that women have higher serum leptin concentrations independent of total body fat mass or percent fat [39, 40]. Compared to older men, higher serum leptin concentrations in older women were not fully explained by visceral and subcutaneous adipose tissues [41, 42]. The protective effect of moderately elevated serum leptin levels on all-cause mortality and CVD-related mortality among older women also suggests that differences in effects of hormones on physiology may be one of the several explanations for gender disparity in mortality rates.

Some studies found positive associations between elevated serum leptin and the risk factors for cardiovascular disease among healthy and diabetic individuals [9, 10, 13, 21, 23, 43]. However, others have shown that elevated leptin reduces cardiomyocyte lipotoxicity by promoting fatty acid oxidation [44, 45] and provides a compensatory role for the reduced cardiac output and improves survival [45]. Leptin has receptors on cardiac myocytes, which exerts direct antihypertrophic effects in heart by stimulating nitric oxide signaling [46]. Levels of serum leptin may have protective effect on cardiac function. Impaired leptin signaling, as in state of hyperleptinemia [47, 48], leads to development of left ventricular hypertrophy [46], and induces chronic oxidative stress in endothelial cells and activates atherogenic process *in vitro* models [17]. Obesity has been consistently linked with the risk of developing cardiovascular disease and heart failure [8, 10], however several previous studies indicated association between overweight and obesity and increased survival in patients with heart failure, which is known as ‘obesity paradox’ [8]. Leptin is secreted by adipose tissue [41] and has been shown to be positively associated with BMI [25] and percent body fat [3, 41]. The protective role of obesity in survival among patients with heart failure may be explained by direct antihypertrophic and antiatherogenic effects of leptin in heart [45, 46].

Although serum leptin has been linked to proinflammatory cytokines, the association of leptin with all-cause mortality and CVD-related mortality was significant after controlling for inflammatory factors, suggesting that the protective effect of leptin may be independent of the proinflammatory state. This may be due to the presence of functional leptin receptors on endothelial cells [46, 49]. Through its receptors, leptin causes antihypertrophic effect [46], coronary vasodilation, activates endothelial progenitor cells and prevents lipid accumulation [44, 45, 49].

The strength of this study includes the longitudinal design of Health ABC study with 10 years of follow-up. HABC’s extensive information on demographic, clinical and biochemical variables made it possible to control several potential confounders such as physical activity and inflammatory markers. There are several limitations of this study. Although there may be some differences in leptin levels by race, the sample size did not allow for stratification beyond gender. However, all the analyses were adjusted for race. The Health ABC study only includes white and black participants, so it was not possible to explore these associations among other racial groups. Also, the participants of this study were well-functioning older adults at the baseline, so the findings may not be generalized to older adults with disability or other age groups.

In conclusion, the results of this analysis suggest that moderately elevated leptin levels may be protective for CVD-related mortality in older women. Among men, serum leptin is not associated with reduced risk of all-cause and CVD-related mortality after controlling for body fat and cytokines. The current finding of an inverse association of serum leptin with CVD-related mortality, independent of body fat and inflammatory markers, among older women should be explored in other research studies.
